# The Local-Balanced Model for Improved Machine Learning Outcomes on Mass Spectrometry Data Sets and Other Instrumental Data

**DOI:** 10.1007/s00216-020-03117-2

**Published:** 2021-02-13

**Authors:** Heather Desaire, Milani Wijeweera Patabandige, David Hua

**Affiliations:** 1 Department of Chemistry, University of Kansas, Lawrence, Kansas 66045, USA

**Keywords:** Software, Genomics / Proteomics, mass spectrometry, machine learning, imaging, glycoprotein

## Abstract

One unifying challenge when classifying biological samples with mass spectrometry data is overcoming the obstacle of sample-to-sample variability so that differences between groups, such as between a healthy set and a disease set, can be identified. Similarly, when the same sample is re-analyzed under identical conditions, instrument signals can fluctuate by more than 10%. This signal inconsistency imposes difficulties in identifying subtle differences across a set of samples, and it weakens the mass spectrometrist’s ability to effectively leverage data in domains as diverse as proteomics, metabolomics, glycomics, and imaging. We selected challenging data sets in the fields of glycomics, mass spectrometry imaging, and bacterial typing to study the problem of within-group signal variability and adapted a 30 year old statistical approach to address the problem. The solution, “local-balanced model,” relies on using balanced subsets of training data to classify test samples. This analysis strategy was assessed on ESI-MS data of IgG-based glycopeptides and MALDI-MS imaging data of endogenous lipids, and MALDI-MS data of bacterial proteins. Two preliminary examples on non-mass spectrometry data sets are also included to show the potential generality of the method outside the field of MS analysis. We demonstrate that this approach is superior to simple normalization methods, generalizable to multiple mass spectrometry domains, and potentially appropriate in fields as diverse as physics and satellite imaging. In some cases, improvements in classification can be dramatic, with accuracy escalating from 60% with normalization alone to over 90% with the additional development described herein.

## Graphical Abstract

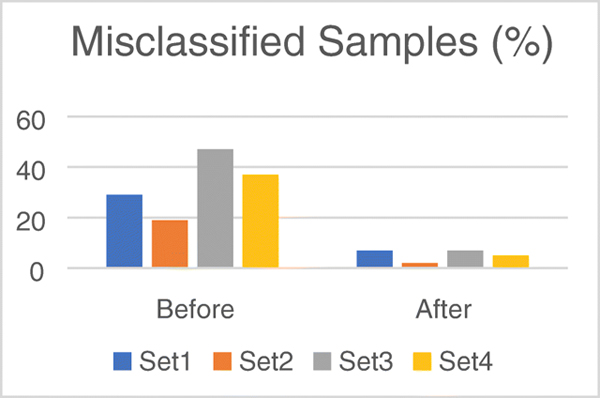

## INTRODUCTION

Because of the power and potential of machine learning, mass spectrometry experts are increasingly interested in using the tools in this field to analyze their data. For example, using DESI-MS data and various supervised machine learning techniques, researchers identified the ethnicity, age, and gender of individuals based on fingerprints [1]. In another example, common infectious bacteria were discriminated without the need for a (slow) culturing step by combining MALDI-MS data acquisition at the single-cell level and machine learning using a fully convolutional network [2]. More recently, individual neural cells could be correctly classified by combining mass spectrometry and boosted decision trees [3]. In each of these cases, among many others, advances in analytical science are achieved that would not have been possible without the marriage of mathematical tools with mass spectrometry. Continued efforts at the interface of mass spectrometry and machine learning have the potential to positively impact both fields.

In addition to using existing machine learning methods, the field of mass spectrometry can benefit by identifying knowledge gaps and solutions where additional math tools could improve analyses. We recently designed a novel supervised machine learning tool, the Aristotle Classifier; this classifier was built with mass spectrometry data in mind, and its unique scoring algorithm has shown promise for various data analysis problems using mass spectrometry data, including: biomarker discovery [4], bacterial typing [5], and MALDI-imaging applications [6]. The classifier has an embedded feature selection function [4], which allows users to include thousands of different m/z’s in the data sets [5, 6]. The classifier more heavily weights the features that better separate the disease state and the healthy state [4], while completely ignoring features that are not useful for discriminating the data. Furthermore, the classifier was designed to be effective on data sets with fewer samples; [4, 5] this aspect is important because mass spectrometrists often test small pilot sets of clinical samples in preliminary biomarker studies. With large feature numbers and small sample sizes, overfitting is usually a major concern. The classifier effectively avoids overfitting, even under these challenging conditions (of high feature number low sample number.) It’s chief benefit, of not overfitting data even in challenging cases, sometimes also becomes its chief limitation: In some cases, the classifier underfits the data, and a different model may provide better accuracy.

In the work described herein, we seek to improve upon the capabilities of the Aristotle Classifier and address another data science problem that mass spectrometrists frequently face: How can one best classify samples with noisy data, where the variation due to the target difference is hard to detect? In mass spectrometry, the signal differences between two groups, a healthy group and a diseased group, for example, can be overshadowed by biological variability, variance in the instrument response, or unintended changes introduced by sample-preparation steps. To date the problem of signal variability, with respect to instrumentation, has been addressed most often by focusing on normalization methods. In depth studies of the best normalization practices for various sub-disciplines of mass spectrometry have been conducted, and guidelines are available for the fields of metabolomics [7], proteomics [8], glycomics [9, 10], and imaging [11]. Any given normalization method has strengths and weaknesses, and no single method is optimal in all cases. As normalization still does not optimally resolve the problem of signal variability in all cases, new approaches are still being considered. State-of-the-art studies even include the development of normalization [12] and peak-recognition functionality [13] using tools from the machine learning field. While developing optimal normalization methods will certainly benefit mass spectrometry as a whole, and in particular, the subset of mass spectrometry studies where machine learning is conducted on the data, we endeavored to address the problem of inconsistent within-group variability in a different way, by combining two different concepts, a local model approach, which is well-known technique in the machine learning field, and a novel technique to address class imbalance. (Class imbalance is the problem of having lots of one type of sample, such as a healthy control, and few of another type, such as a disease state. Most machine learning tools work optimally when the two sets of data have equal number of samples.) We combine the concept of the local model, with a new idea for addressing class imbalance, into a single proposed solution called the local-balanced model. This new technique ameliorates the problems introduced from signal variability by generating a unique trained model for each unknown sample. As demonstrated herein, classification with the local-balanced model is a complementary method that can provide a performance enhancement over using a base classifier on normalized data. In some cases, optimal performance can be obtained when both normalization and the local-balanced model are used together.

The local balanced model is most similar to the existing strategy of combining of a k-Nearest Neighbors (kNN) classifier with a second classifier, such as Support Vector Machine (SVM) or Naïve Bayes. The most relevant combination of kNN and SVM, where kNN is used to define the training data for a local SVM model, has been around since at least 2006 [14, 15]. A modification of this approach is taking off as a way to provide scalability to SVM, which becomes intractably slow when the number of labeled samples is large [16]. The combination of kNN and Naïve Bayes classifiers is also known [17], and this pairing has been implemented since the early 1990s (18). In this case, using kNN to select instances to build a classifier improves Naïve Bayes by weakening the attribute independence assumption of the Baysian classifier [19]. Importantly, none of these cited examples, or other examples of implementing a local model apply the technique to mass spectrometry data.

The two innovations described herein, then, are to apply a local model to mass spectrometry data and to improve its classification accuracy by balancing the subsets used for training. While local model strategies are known inside the field of machine learning, and have been around for about 30 years, they have never been applied to mass spectrometry data. Furthermore, in all the examples we have studied where kNN and another classifier are combined, no effort is made to balance the number of samples from each class in the training group. Rather, the N nearest training samples, relative to the sample to be classified, are used. By contrast, in the *local-balanced model* paradigm, the number of samples in each class are specified as a tuneable parameter. Therefore, a model may contain equal number of labeled samples from each class, even though the Euclidian distances for the most different sample in one class may be substantially farther away from the test sample than the least-nearest neighbor included from the other class. The local-balanced model concept, therefore, is unique from prior work in the field of machine learning, and the general adaptation of a local model is new in the field of mass spectrometry.

We successfully applied the local- balanced model to five diverse data sets using two different classifiers. Three of the data sets encompass exclusively mass spectrometry data; the other two are non-mass spectrometry examples where variations due to instrument response are also potentially embedded in the output data. In almost all cases, using this approach does better than existing machine learning methods that do not include a local-balanced model. In some cases, the improvements are spectacular. We also show examples where using a local-balanced model does not substantially improve the accuracy of the base classifier. Including these counterexamples is meant to be instructive for readers, so they can better understand the circumstances in which a local balanced model can improve the overall accuracy of a given model. The local-balanced model, which was developed specifically for mass spectrometry data, but shown here to be applicable to multiple types of instrument-generated data, has the potential to offer scientists in diverse fields the ability to classify challenging data sets with highly enhanced accuracy.

## EXPERIMENTAL

### General testing conditions.

Unless otherwise stated below, the following procedures apply to all classifications conducted herein. All machine learning was done in R Studio, using R version 3.5.1. SVM was conducted using the e1071 package. The base Aristotle Classifier results were obtained using the previously published algorithm [5]. For all local-balanced model classifications, the algorithms used are those provided in Supplementary Information (ESM): Local.Balanced.Aristotle.Classifier.txt and Local.Balanced.SVM.txt. The means of calculating accuracy and AUC are described with respect to each classifier, below. G-mean and F-measure for the minority class were calculated as described in reference 20.

When the local-balanced model, provided in ESM, is used for classification, certain input data are required. The data set to be classified, in the orientation required for the method being implemented, is input, along with the data set dimensions. The identities of the training samples for the two groups being classified (“Low” and “Hi”) need to be specified, along with the desired samples to be tested. Aside from this basic input information, the only parameters optimized in these studies were the sizes of the training sets for the local-balanced model. The training set size is a manually adjustable value that appears in the lines of code defining “LowTrain” and HiTrain”, approximately line 40. Naturally, researchers could choose to additionally optimize the models, by modifying the hyperparameters in SVM, for example. Since the goal of these studies was not to achieve the best-possible outcome, but rather to compare a standard set of conditions – with and without a local-balanced model – we did not adjust any hyperparameters beyond those specified herein.

### Considerations for SVM.

All data sets were processed so that the samples were in rows and the features were in columns. Before all SVM classifications, data sets were scaled using the embedded “scale” command in R unless otherwise noted. The test sample was never included as part of the training set: for base SVM classifications, a leave-one-out strategy was employed, and during testing with the local-balanced model, the test sample is automatically excluded from the training set in the code provided. The accuracy for all SVM classifications was determined without considering probabilities, as this method generally produced optimal results on these data sets. The assigned class identities (either Group 1 or Group 2) were compared to the correct class identity for each sample, and the overall accuracy was calculated by determining the percentage of the correctly assigned samples. The AUC (area under the receiver-operating curve) was determined using the probabilities for each sample; it was calculated using the package, pROC [21].

### General considerations for the Aristotle Classifier (AC).

All data sets were processed so that the features appeared in rows and the samples appeared in columns. For all calculations, the test samples were always excluded from the training set. The class identity was determined by the sign of the final Result, as described previously [4]. When the sign of the final Result was positive, the sample was assigned to Group 1. When the sign of the Result was negative, the sample was assigned to Group 2. The overall accuracy of the model was determined by calculating the percent of samples that were correctly assigned to their respective groups. To determine the AUC for these experiments, the pROC package was again employed, and the raw numerical scores from the Result vector were used. Unless otherwise specified, k was set to 500.

### Data set 1. IgG.

**Figure 1.**
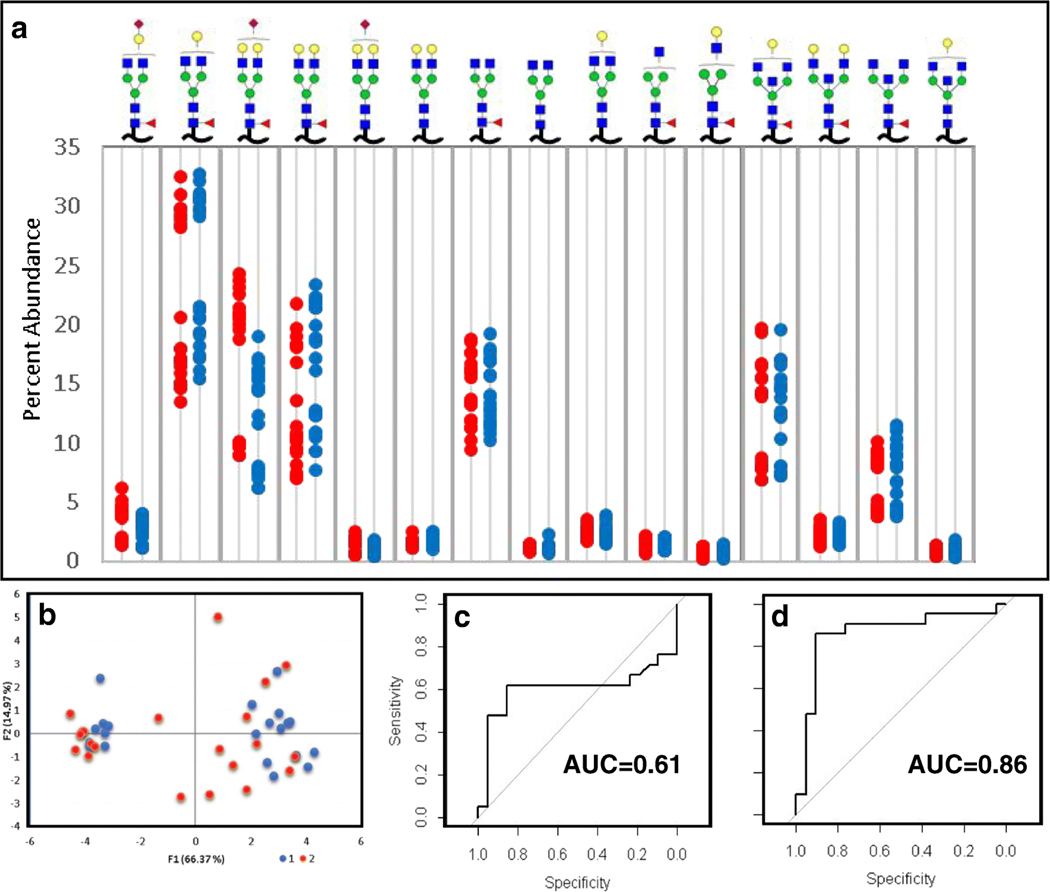
Initial characterization of the IgG1 data set. A) The percent of each glycopeptide for 21 samples of native IgG1 (red dots) and 21 samples of modified IgG1 (blue dots.) Glycopeptide compositions (top) are proposed based on the high-resolution mass and MS/MS data. B) PCA plot, which shows the main variability in the data is not related to the change in glycosylation. C) Receiver-operating curve (ROC) for supervised classification using the Aristotle Classifier. D) Receiver operating curve (ROC) for supervised classification using Support Vector Machine (SVM.)

LC-MS data of 42 IgG1 samples, where 21 samples had a native glycosylation profile and 21 samples had a modified profile, were available from a previous experiment [4]. The samples with a modified glycosylation profile contained a mixture of native IgG and IgG that had been partially desialylated, by neuraminidase A treatment. It was estimated that the samples were approximately 20% disialylated [4]. The IgG1 glycopeptides in these LC-MS files were identified and quantified in the same manner that the IgG2 glycopeptides had been identified previously [4]. Specifically, a known set of IgG1 glycopeptides was searched for [22], and their presence was verified manually by assigning both high-resolution MS and MS/MS data [23]. After this initial validation on one of the LC-MS files, the glycopeptides in each subsequent LC-MS file were confirmed by high-resolution MS data and retention time. Fifteen glycopeptides from IgG1 were detected in all 42 samples, so these were the glycopeptides whose abundances were included in the data set. The raw abundance of each ion, at the 50% abundance of extracted ion chromatograms, was acquired and transformed into a percent, so that the total IgG1 glycopeptide signal for each sample added to 100%, as previously described [24]. Figure 1A shows all the glycoforms’ relative abundances for each of the 42 samples.

For classification with the Aristotle Classifier (AC), the data set of (unscaled) percents were used. When the local-balanced model was incorporated with AC, the training sets included seven samples from each class. For classification with SVM, the training sets included nine samples from each class; these values were determined through optimization.

### Data set 2: MALDI-Imaging.

This data set is described in a prior report [6]. It contains 461 samples; each sample is a pixel in an imaging experiment, and 3500 features; each feature corresponds to the ion counts present in one of 3500 equal-spaced bins along the mass range of *m/z* 210.3 to *m/z* 994.5. The mass spectral data were acquired in the negative ion mode, and, based on the mass range and the ionization mode, the features are assumed to represent endogenous metabolites, mainly lipids, from cancer spheroids. The difference between the two classes is that one class represents pixels from a spheroid that had undergone chemotherapy treatment with cetuximab, while the samples from the other class originate from a spheroid that had not undergone treatment. More information about data acquisition can be found in reference 25.

For classification of the MALDI-Imaging data, the MS signal of each sample had already been normalized so that the total ion counts for each sample summed to 1.0 [6]. For the local-balanced model with AC, 20 samples were selected for each class for training. For the local-balanced model for SVM, no subset tested outperformed the results for SVM-base, so all the samples available – except the test sample – were used in the local-balanced model.

### Data Set 3: Satellite.

The Satellite data set contains a total of 6435 samples and 36 features. In this case, the samples correspond to different geographic locations, and the features correspond to different light readings. The data were acquired from the UCI Machine Learning Repository [26]. Six classes were present in the data; for the experiments conducted herein, the target class was “4” (damp, grey soil), and all other samples comprised the non-target class. This specific target class was chosen so the results could be benchmarked against a landmark study comparing various decision-tree-based classifiers; in that work the target, class 4, was also selected [20]. For this data set, optimal conditions for the local-balanced model included 10 samples from the minority (target) class and 18 samples for the majority class for both classifiers.

For the train/test experiment on the Satellite data set, the last 965 samples were reserved for a test set, leaving the first 5470 samples to be used for training. Classification was performed on the test data with both base models (SVM and Aristotle Classifier), using only the first 5470 samples in model training. Classification was also performed on the test data using both local-balanced models. During the train/test experiments, the group sizes were re-optimized since the size and composition of the training set was changed. As before, optimization proceeded by maximizing the AUC on the training data. The optimal group size for both SVM and the Aristotle Classifier were 10 samples for the minority class and 18 samples for the majority class.

The “Hill-Valley” data set was also acquired from the UCI Machine Learning Repository [27]. This data set has 1212 samples and 100 features; the nature of the samples and features are described in more detail below. One important aspect to note here: Two different versions of the “Hill-Valley” data set are available. We exclusively used the harder-to-classify data set, the one that had noise incorporated into it. The results below, therefore, should not be compared to classifications of the easier-to-analyze noise-free data set, which is more commonly studied.

For this data set, the data were scaled using the embedded function in R studio, and the scaled version was used for all classifications with both classifiers. For SVM, the local-balanced model performed optimally with 90 samples from each class; for AC, the optimal number of samples per class was 8. In addition to the classification on the original set of features, a second local-balanced model classification was performed using the Aristotle Classifier on a larger feature set. It included the original set of 100 features, along with all possible ratios of these features. The code to automatically generate the expanded feature set, with a non-redundant list of all the possible ratio pairs, is provided in ESM. The file is labeled: Ratio.Builder.txt. Due to the larger feature set size, k was set to 100 for this classification.

For the train/test experiment on the Hill-Valley data set, the last 100 samples from each group were reserved for a test set, leaving the first 1012 samples to be used for training. Classification was performed on the test data with both base models (SVM and Aristotle Classifier), and both local-balanced models were also used to classify the test data. For the SVM classification, the data set containing 100 features per sample was used. For the Aristotle Classifier, the expanded feature set, of 5050 features per sample, was used. During the train/test experiments, the group sizes were re-optimized since the size and composition of the training set was changed. As before, optimization proceeded by maximizing the AUC on the training data. The optimal group size for SVM was 100 (for both groups). The optimal group size for the Aristotle Classifier was 8, also for both groups.

The final data set studied herein is the ”MicroMass” data set. It has been described previously [28], and is available from the UCI machine learning repository. It contains a training set of 571 samples of bacterial proteins that had been subjected to MALDI-MS; each sample’s genus and species is identified. For this study, the Gram positive bacteria (from the genera: QWP, WNJ, QBG, and RTO) were compared to the Gram negative bacteria (from the genera: JNH, NYV, BUT, EMD, and AUG.) For each sample, 1300 features were provided, where each feature corresponds to the ion counts detected in a particular mass range during MALDI-MS analysis. Some of the 1300 features had no ion abundance for any of the 571 samples, so they were removed prior to analysis. The classifications were performed on the remaining 1082 features, using the raw MS abundances from these features. When the local-balanced model was optimized on the training set, SVM was the classifier, and a total of 130 samples were selected for each group.

In addition to the 571-sample training set, a separate test group is available for this data set. The test set contains an additional 360 samples; not all the test samples could be used in this study because some of them were mixtures of two different Gram types. Therefore, prior to analysis, the test group was partitioned so that all the samples in the group were either 100% Gram negative or 100% Gram positive: All test samples that were from a single genus/species were used along with all samples that contain a mixture of different bacteria from the same Gram type. Thus, a total of 248 test samples were used to evaluate model performance after all optimization was completed on the training data. Again, 130 training samples were used for each group in the local-balanced model.

## RESULTS AND DISCUSSION

### Motivation for the work.

The data in Figure 1 represent a challenging classification problem that sparked the development of the local-balanced model. Figure 1A shows glycopeptide abundances for 42 different IgG1 samples; half the samples (red dots) contained one glycosylation profile, and the other half of the samples (blue dots) contained a slightly different profile. The samples were either unmodified IgG, or IgG that was 20% deficient in sialyalated glycoforms. We generated the sialylation-deficient IgG in-house through a glycosidase reaction [4]; the two groups of samples were developed to challenge existing machine learning tools: the glycosylation variability between the two sample sets was small and only imparted on a subfraction of the glycopeptides that were quantified. As shown in Figure 1A, which displays the glycopeptide abundances for all 15 glycopeptides, measured for all 42 samples in the data set, no single glycopeptide’s abundance is useful in discriminating the two groups. Furthermore, as shown in Figure 1B, the PCA plot indicates that the variability between group 1 and group 2 is not the main variability in the sample set; therefore, distinguishing whether a new sample was from group 1 or group 2 would be challenging.

Poor classification indeed results when these data are subjected to various classifiers (using a leave one out validation strategy). When the Aristotle Classifier is used to classify the samples, a disappointing result is obtained. The receiver-operating curve (ROC) is in Figure 1C; the area under the curve (AUC) is an uninspiring 0.61. Could a different classifier have done better? In Figure 1D, the ROC plot for a model built with an SVM (Support Vector Machine) classifier is shown. This classifier certainly performed more admirably, but the AUC was still below 0.9, and five of the 42 samples remained misclassified.

Since our ultimate driver in studying this sample set was to use it as a testbed to improve upon the classification tools available for mass spectrometrists, we next set out to identify an analysis approach that would offer better classification accuracy than what had been achieved in Figure 1. The challenging aspect of this sample set was introduced by the inconsistent instrument response; in the PCA plot, for example, the samples that partition on the left side of the plot generally had higher ion counts than the samples on the right side of the plot. It should be noted that when these data were classified, they had already been normalized using the standard approach in the field of reporting the glycan abundances as a fraction of 100% of the total glycan abundance [10, 24]. So this case represents an example where the normalization strategies already in use in the field are insufficient to provide good classification outcomes, even though the problem with classification is mainly related to inconsistent ion signal. We hypothesized that a one-model-for-one sample approach that only uses labeled (known) samples that are highly similar to the sample being classified would do much better than a more conventional strategy, where all the labeled samples are used to generate a single model to classify every unlabeled sample. In the field of machine learning, this concept fits under the umbrella of a “local learning” strategy [29]. While this method has not be applied in the field of mass spectrometry before, the rationale for pursuing this approach was that the smaller model, with test samples more like the known samples, would evade the issue of experiment-induced variability that was most likely responsible for the poor classification results. To test the hypothesis, we wrote a software script to automatically select which samples to use for training; the code is available in ESM. Training samples are first selected based on their similarity to a test sample, and that subset of training samples is used to build a local-balanced model for a single test sample. Selecting a subpopulation of labeled samples is straightforward: the Euclidian distance of each sample (in n-dimensional space, where n is the number of features) is used to select two sets of training samples – one from each class- to use for training a classifier to classify the sample of interest. Note: samples from each class are independently selected, based on an input number of desired samples for that class. This is a manually adjusted parameter near line 40 in the code.

After sample selection, a local-balanced model can be generated for each sample to be classified using any desired classifier. Two different classifiers were selected for the experiments herein: a Support Vector Machine (SVM) classifier was chosen because it is well known for its general utility for biomarker studies, where feature space is often greater than the number of collected samples [30]. The Aristotle Classifier (AC) was also tested; we have developed it specifically for classifying biomarkers that are identified based on mass spectrometry data.

In applying the local-balanced model strategy, researchers must decide how many labeled samples should be included for model training. There is no one best answer to this question; optimal results vary from one data set to the next. The number of samples selected is thus a tuneable parameter. In the work described herein, we optimized the sample numbers to achieve an optimal area under the receiver-operating curve (AUC), as this is a well-recognized predictor of model performance [31]. It would certainly be possible to choose the number of labeled samples that lead to optimal overall accuracy or the highest recall of the minority class or any other attribute that is desired.

### Test Data.

**Figure 2.**
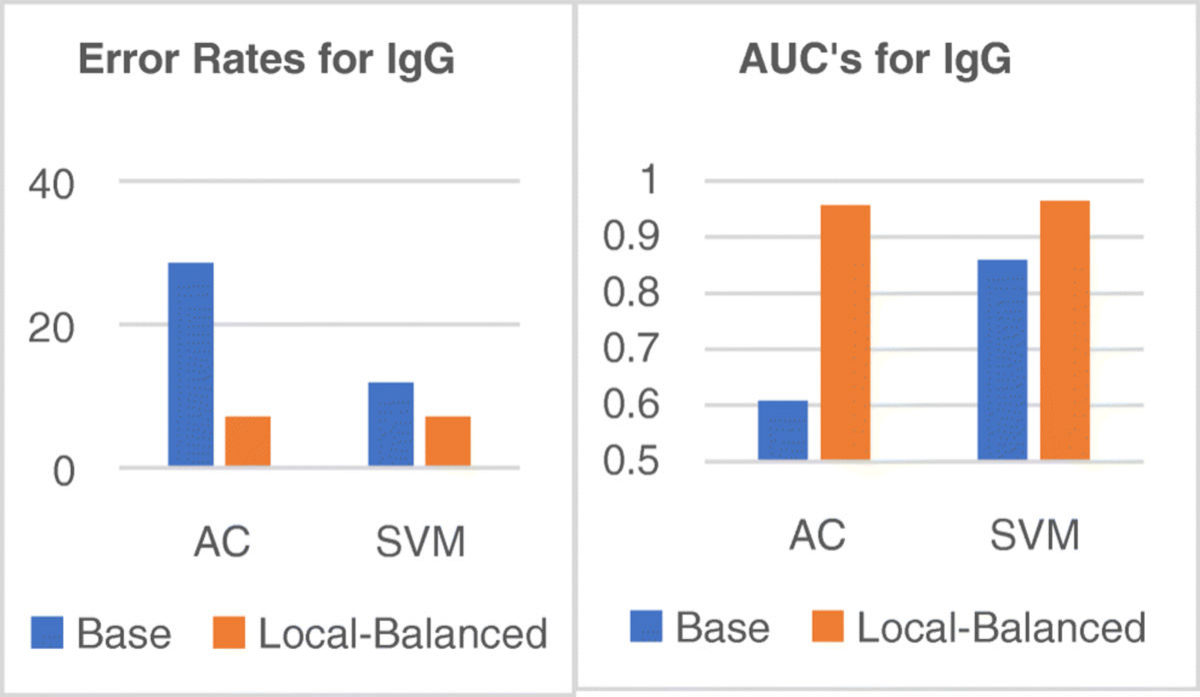
Error rates and AUC (area under the curve) for the IgG data set. The error rates (expressed in percent error) drop precipitously between the base classifier and the local-balanced model. Likewise, the AUC, a key measure of model performance, increases when the local-balanced model is incorporated with either classifier. SVM=Support Vector Machine; AC = Aristotle Classifier.

Figure 2 compares the outcomes of either using a local-balanced model or not when classifying the glycopeptide samples shown in Figure 1; additional details describing how the local-balanced model was implemented are provide in the Experimental section. This figure shows that the two classifiers were affected to different degrees by including the local-balanced model, yet both benefitted from its implementation. The Aristotle Classifier showed a dramatic improvement in overall accuracy. Without implementing a local-balanced model, 14 samples were incorrectly classified, but that number dropped to only three misclassifications with the optimal implementation of a local-balanced model. By contrast, the SVM classifier showed a smaller improvement in overall accuracy; five samples were mis-classified before the local-balanced model was implemented, and three were misclassified after. The same trend is observable when one considers the AUC in each instance. The AUC for the base Aristotle Classifier was not very good (0.61), while the base SVM approach had an AUC of 0.86. When the local-balanced model was implemented, both methods’ AUC’s improved to 0.96. This classification result – 93% accuracy and an AUC of 0.96 – is a prodigious achievement, considering the PCA data in Figure 1B, which foretells that no effective classification would be achievable.

Next, we applied the local-balanced model concept to other data sets, with the goal of learning more about its utility and general applicability. The second sample set contained MALDI imaging mass spectrometry data that had been recently classified using the Aristotle Classifier [6]. This sample set contained metabolomics data from two spheroids that either had (group 1) or had not (group 2) undergone chemotherapy treatment with the monocolonal antibody, Cetuximab. The data were acquired after 24 hours of treatment, an early time point that had been shown to occur prior to the major metabolomic changes that the spheroids would undergo upon prolonged treatment exposure [25]. Therefore, the samples at this early time point are rather similar. A total of 461 examples were available, each representing a pixel from a spheroid that had originated either from the control or the treatment group.

As shown in Table 1, the sample set was not optimally classified using the Aristotle Classifier alone, which was the classification strategy originally used on these samples [6]. Only 81% of the pixels could be correctly assigned to their correct group (control or treatment), and the AUC was 0.910. When the local-balanced model was combined with the Aristotle Classifier, a substantial improvement was observed: the accuracy shot up to 98%, and the AUC was 0.994. This performance was approximately equivalent to the performance produced by SVM, either with or without the local-balanced-model component. In both cases, the accuracy was 98% and the AUC was 0.999.

After these studies, we endeavored to determine the possibility of using of the local-balanced model concept to improve classification accuracy of data outside the domain of mass spectrometry. Could this novel implementation of the local model concept, using balanced numbers of samples in the training sets, provide a general benefit to the machine learning field compared to the approaches that are currently implemented for classification problems? To answer this question, we describe results from two example data sets that are difficult to classify accurately using conventional methods. The first example contains satellite imaging data, where the goal is to predict the presence of a particular feature based on a numerical rendering of the light readings received from the satellite [26]. This prediction is challenging, in part, because of the class imbalance in the data. Only about a tenth of the examples in the data set correspond to the target class. Furthermore, it represents an example where the data to be classified are instrument readings, which may introduce variability in the data set due to inconsistent response.

In this case, the base SVM classifier (without implementing a local-balanced model) was unable to correctly assign a single target sample to the target class. Rather, the model that is generated is the dreaded “majority state classifier”, where every example gets assigned to the majority class. While one may note that this model results in an accuracy of 90%, since 90% of the species are in the majority class, such a model is completely useless, as it has no ability to discriminate between the target and non-target examples. When a local-balanced model is incorporated with SVM, much better performance is realized. Two thirds of the minority class examples are correctly predicted, and the AUC is substantially improved. The accuracy is also increased, although an increase in accuracy of about 5%, as shown in Table 2, substantially under-emphasizes the improvement in the model. For imbalanced data sets, such as this one, model accuracy is sometimes not even reported; instead the G-mean, F-measure of the minority class, and AUC are preferable indicators of the model’s performance [20]. These terms do a better job of reflecting model performance while accounting for the disparity in sample size in the two groups. Table 2 shows that by using these accepted standards, an astonishing improvement was obtained when comparing the base SVM method to the local-balanced model plus SVM. The G-mean, a term that can range from zero to one, goes from zero to >0.8.

Interestingly, SVM with local-balanced model was not the best performing approach for classifying these data. The local-balanced model with the Aristotle Classifier had a slight edge. Its AUC was higher (0.967 vs 0.957); its G-mean was higher (0.83 vs 0.80) and its F-measure was higher (0.73 vs 0.72). While these results are better than either attempt with SVM, the data in Table 2 also show that the local-balanced model component was necessary to achieve this performance. Without that component, the Aristotle Classifier achieved less impressive results, including an AUC of 0.89.

While the local-balanced model was successful at improving classification performance for both the AC and SVM classifiers, one might like to know how these results compare to the large array of other possible ways of classifying these data. For example, decision tree-based methods are often used in cases where a large class imbalance exists [20], and these methods, coupled to undersampling techniques, frequently outperform classifiers like SVM for problems of this type. Fortunately, 15 different methods optimized specifically for class imbalance learning have been previously used to study this data set [20]. These studies included head-to-head comparisons of some of the best strategies in use today that combine undersampling and decision trees, including SMOTE, AdaBoost, etc. Against these 15 strategies, the best performer on this data set, which was an oversampling approach combined with Random Forests, gave an AUC of 0.962, a G-mean of 0.782, and an F-measure of 0.689 for the minority class. The results achieved by coupling the local-balanced model with the Aristotle Classifier are better. Furthermore, the full extent of the potential of a local-balanced model is not yet tapped; for example, no effort was made to further optimize the SVM classifier using a cost-sensitive model, which would likely be useful for squeezing out more correct assignments for the minority class.

**Figure 3.**
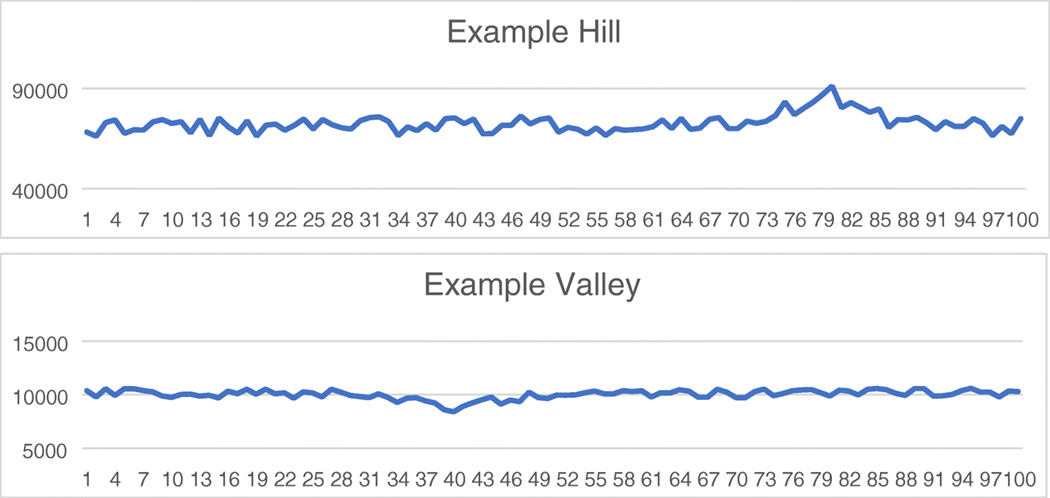
Examples from the Hill-Valley data set. These graphs show data for two different samples: a hill (top) and a valley (bottom). Each graph is a continuous line connecting the 100 numeric features for each sample, which are plotted in order along the X axis. The Y axis, in arbitrary units, could be considered “instrument response”.

The next data set in this study, “Hill-Valley,” is renowned for its classification difficulty. In this case, 1212 examples are available. For each example, a simulated instrument signal is generated that either goes up or down over the course of 100 measurements. Instrument response for 100 data points (100 features) are provided, which, when plotted along an x axis, either generate a “hill” or a “valley” because a graph of the feature values either have a recognizable peak or trough. An example of a “hill” and a “valley” from this data set is shown in Figure 3. This data set is challenging to classify because the data contain noise (random fluctuations), and the instrument response is inconsistent: the magnitude of the response changes, and the target aspect, the hill or valley, appears at varying widths and heights, and at varying locations across the 100-point interval. Furthermore, no single feature would provide the correct answer; rather the class (hill or valley) is only identifiable if a model accounts for how the features relate to each other. Published examples of classification using this data set are rare in the literature, likely because few methods can accurately classify the data. In a published thesis, several classification methods were used, and accuracy hovered in the low 60 percents [32]. In the peer reviewed literature, both SVM and kNN were assessed on this data set; without dimensionality reduction, the classifiers achieved <60% accuracy [33].

Table 3 shows how the AC and SVM classifiers perform compared to the aforementioned benchmarks, with and without incorporating a local-balanced model. We also studied the impact of modifying the feature set when using the Aristotle Classifier. Considering only the first (original) feature set, one can easily infer that the Aristotle Classifier is the wrong model for these data. Neither the base AC nor the local-balanced model-enhanced classifier did much better than flipping a coin would have done to predict which examples were “hills” vs “valleys”. This outcome is not surprising to those who are familiar with the underlying scoring mechanism present in this classifier. Each feature is considered individually, and such an approach would not be effective with a data set like this, where the correct classification must be inferred by considering the features’ relationships to each other. This limitation of the classifier can be overcome by considering ratios of the features, and in the original publication of this method, complete sets of ratios containing two and three features were built and classified [4]. Since this data set has 100 features, it is feasible to consider the complete list of ratios of two features (about 5000 features), but the complete list of all possible ratios of two *and three* features would be unwieldy: approximately 250,000 features. Therefore, we wrote a short script, provided in ESM, that accepts any input matrix and automatically generates a new output matrix that contains ratios of all the pairs of features in the original input matrix. Using this output matrix– containing the original 1212 samples, with each sample now containing about 5000 features – a local-balanced model was again built with the Aristotle Classifier. Since this new feature set addresses the relationships between the different features, it is useful for accurately solving this challenging classification problem. The AUC rockets up to 0.99 and the accuracy climbs to over 93%, as shown in Table 3.

The performance of the SVM classifier on this data set is quite similar to that of the Aristotle Classifier, with the caveat that the SVM classifier already accounts for the feature relationships, so an enlarged feature set containing ratios of the peaks is not needed. The base SVM method is not that effective at assigning the data, but incorporating a local-balanced model with SVM shows a dazzling performance enhancement. Accuracy jumps from 60% to almost 94% and the AUC rises from 0.80 to 0.98. This performance dramatically exceeds all benchmarks that we could identify in the published literature.

The performance enhancements provided by the local-balanced model are dramatic in the last two data sets, and skeptics may wonder whether this is due to overfitting the data. To address this question, we changed the way the classification was done on the Satellite and Hill-Valley data sets. In the new experimental design (for both data sets), the model was built using only the first ~85% of the data, and the last ~15% of the data was reserved as a test set. The optimal group sizes were re-determined with the new training data, using a leave-one-out validation, and the optimized model was then applied to the test data. In both the Hill-Valley and the Satellite data sets, no drop in performance was seen between the train and test data with either classifier. Indeed, the AUC was >0.97 for both classifiers on both data sets. (see ESM Table S1.) This experiment clearly demonstrates that the high-quality results shown here are not due to model overfitting. Rather, they represent an ideal data science strategy for classifying these data.

In considering the experiments presented here in aggregate, one final question remains. Why did the local-balanced model offer significant improvements for the SVM classifier on the two non-mass spectrometry data sets, but little or no improvement on the two examples containing mass spectrometry data? We hypothesized that the answer to this question lie in the data sets selected for study. The first mass spectrometry data set was rather small, so removing training samples offered little benefit. In the second case using MS data, the base model already performed optimally, so no improvement could be made. By contrast, in both the two non-mass spectrometry data sets, many more training samples were present, and the base model performed poorly. We hypothesized that if a mass spectrometry data set with more training examples were studied, and the base SVM model had room for improvement, the local-balanced model may show a performance enhancement over a basic SVM model. To test this hypothesis, we studied one final dataset, “MicroMass”, which contains MALDI-MS data for 571 different bacterial samples.

Does the local-balanced model offer a better classification on this data set? To answer this question, one must first define the classification problem to address. The data set contains MS abundances for 1300 peaks from a total of 20 different species of bacteria from 8 different genera. Furthermore, about 30% of the training samples are from Gram positive bacteria, while the remaining 70% are from Gram negative bacteria. Since the goal was to test the hypothesis that the local-balanced model would outperform the base SVM model when enough training samples (from both classes) are available, the classification problem that would best achieve that goal is determining whether each sample can be classified correctly as Gram negative bacteria vs. Gram positive.

Table 4 shows the results for leave-one-out testing using the base SVM classifier for this problem. In all, 28 of 571 samples were misclassified, and the area under the receiver operating curve was 0.983. By contrast, implementation of the local-balanced model with SVM cut the error rate by a factor of three. Only nine of 571 samples were misclassified, and the AUC improved to 0.998. These data, again, demonstrate that the local-balanced model was more effective.

But was this increase in accuracy due to overtraining or due to a truly better model? Fortunately, this question is readily answerable with this data set because the investigators who collected the data also generated a second independent test set, which provided an additional 248 unseen samples to study. These samples had been generated using the same methods as the training set. Using the model built on the training data, the test samples were classified. The base model misclassified 17 samples, or about 7% of the 248 samples. The AUC for the test set, using the base classifier, was 0.964. This AUC is slightly lower than that for the base SVM model of the training data. The local-balanced model significantly outperformed the base model, misclassifying only 3 of the 248 samples. The error rate was about 1%, and the AUC for this test set was 0.9998. (See Table 4). This example again demonstrates that the local-balanced model truly generates a better model, and its performance enhancement is not due to overtraining.

This example also provides users with more information about the circumstances under which this approach offers a benefit: the method, which removes samples from the training set, is one that has a higher likelihood of outperforming the base model when the training set is adequately sized. “Adequately sized” likely varies from one case to another, but based on this principle, we expect that the approach may be more useful in MS-imaging applications, high-throughput (high sample) problems, like bacterial typing, and large-scale clinical studies; by contrast, pilot biomarker studies, where the sample sets often contain less than 50 samples, would probably benefit less from this approach, particularly when coupled with an SVM classifier.

## CONCLUSION

We studied the problem of inconsistent instrument response in mass spectrometry data and developed a useful solution: the local-balanced model. This method is a small tweak on an old machine learning strategy, the local model, although that strategy has never before been implemented in the mass spectrometry field. Using the local-balanced model, we observed an impressive improvement in supervised classification performance with two different classifiers and multiple data sets. The approach is simple, accomplished with just a few lines of code that are provided in ESM, and it adjusts for inconsistencies introduced by instrumental variation. We demonstrated the approach’s effectiveness on three examples in the field of mass spectrometry and two additional examples outside the field, where instrumental data are used for machine learning. In several cases, the incorporation of a local-balanced model resulted in classifications with AUC’s of >0.95. This performance exceeds existing benchmarks and offers mass spectrometrists and other analytical scientists a new tool to generate better predictions from their existing data.

We fully concede that using a local-balanced model will not offer improvements in every classification problem; we demonstrated the point herein already. If the classifier is performing very well on its own, as was the case with SVM and the mass spectrometry imaging data, the local-balanced model will likely not make it better. Furthermore, if the data set already has a small number of samples in it, then subsetting the training samples may not be useful in generating an improved model. By contrast, the classification problems that will most benefit from incorporating a local-balanced model are those that are currently hard to solve: cases where experimental variation impacts samples differently across a sample set and cases where the characteristics defining a given class label are nonuniform. These types of problems are ideally suited for machine learning with a local-balanced model. Therefore, we expect this innovation to improve classification in multiple domains, including MS analysis for large clinical sample sets, imaging applications, and areas beyond these domains, to other scientific applications that rely on instrumental data, and more broadly, to any domain where training a model with a relatively homogenous, sub-population of samples would provide a superior answer to a classification question.

## Supplementary Material

1686935_Supp_Info4

1686935_Supp_Info3

1686935_Supp_Info2

1686935_Supp_Info1

## Figures and Tables

**Table 1.** Classification Results for Imaging Data

		Aristotle Classifier	SVM	
	base	local-balanced	base	local-balanced*
AUC	0.910	0.994	0.999	0.999
accuracy	80.7%	97.6%	97.8%	97.8%

* No improvements were made with smaller data sets; this local-balanced model includes the full data set.

**Table 2.** Classification Results for Satellite Data

	Aristotle Classifier		SVM		Benchmark*
	base	local-balanced	base	local-balanced	
AUC	0.894	0.967	0.714	0.957	0.962
G-mean	0.758	0.831	0	0.804	0.782
F-meas (minority)	0.337	0.729	0	0.718	0.689
Accuracy	62.8%	94.9%	90.3%	94.9%	n/a

* Benchmark data from ref 18; the best approach of 15 different decision tree based methods is shown.

**Table 3.** Classification Results for Hill-Valley

	Aristotle Classifier			SVM	
	base	L-B#1*	L-B#2*	base	L-B
AUC	0.521	0.588	0.986	0.798	0.975
accuracy	0.534	0.557	0.932	0.602	0.938

* L-B#1 is the local-balanced model on the origianl feature set. L-B#2 is the local-balanced model on an expanded feature set that includes all possible ratios of two features.

**Table 4:** Classification Results for Bacterial Typing w/SVM

	Base Model	Local-Balanced
Experiment	Train (LOO)	Train (LOO)
AUC	0.983	0.998
# samples	571	571
**# errors**	**28**	**9**
Accuracy	0.95	0.98
	Base Model	Local-Balanced
Experiment	Test	Test
AUC	0.963	>.999
# samples	248	248
**# errors**	**17**	**3**
Accuracy	0.93	0.99
