# Supplementary material for: The Local-Balanced Model for Improved Machine Learning Outcomes on Mass Spectrometry Data Sets and Other Instrumental Data
Source: Anal Bioanal Chem. Author manuscript; Available in PMC 2022 Mar 1. (PMC8516084; doi:10.1007/s00216-020-03117-2)
Supplement: Supplementary file 1 [file NIHMS1686935-supplement-1686935_Supp_Info4.docx]

Table S1: Train/Test Results for the local-balanced mdel

	Satellite data set			
	Aristotle Classifier		SVM	
	LOO^	Test	LOO^	Test
# Samples	5470	965	5470	965
AUC	0.968	0.979	0.951	0.972
Accuracy	0.947	0.956	0.949	0.944

^LOO=Leave One Out; these are the training results.

	Hill-Valley data set			
	Aristotle Classifier*		SVM	
	LOO^	Test	LOO^	Test
# Samples	1012	200	1012	200
AUC	0.98	0.993	0.969	0.974
Accuracy	0.92	0.975	0.936	0.935

*Results for L-B#2 are shown. As in Table 3, this data set includes the all the original features and their ratios.
